# Exploring the Feasibility and Effectiveness of a Childcare PhysicaL ActivitY (PLAY) Policy: Rationale and Protocol for a Pilot, Cluster-Randomized Controlled Trial

**DOI:** 10.3390/ijerph16224400

**Published:** 2019-11-11

**Authors:** Patricia Tucker, Molly Driediger, Leigh M. Vanderloo, Shauna M. Burke, Jennifer D. Irwin, Andrew M. Johnson, Jacob Shelley, Brian W. Timmons

**Affiliations:** 1 School of Occupational Therapy, Faculty of Health Sciences, University of Western Ontario, London, ON N6A 3K7, Canada; mdriedig@uwo.ca; 2 Child Health Evaluative Sciences, The Hospital for Sick Children, Toronto, ON M5G 0A4, Canada; leigh.vanderloo@sickkids.ca; 3 School of Health Studies, Faculty of Health Sciences, University of Western Ontario, London, ON N6A 3K7, Canada; sburke9@uwo.ca (S.M.B.); jenirwin@uwo.ca (J.D.I.); ajohnson@uwo.ca (A.M.J.); jshelle6@uwo.ca (J.S.); 4 Faculty of Law, University of Western Ontario, London, ON N6A 3K7, Canada; 5 Child Health and Exercise Medicine Program, Department of Pediatrics, McMaster University, Hamilton, ON L8S 4K1, Canada; timmonbw@mcmaster.ca

**Keywords:** physical activity, childcare, policy, preschooler, toddler, sedentary time, protocol

## Abstract

*Background*: Young children are prone to low levels of physical activity in childcare. This environment, inclusive of equipment, policies, and staff, has been identified as influencing young children’s activity behaviours. To date, no study has examined the feasibility and effectiveness of such policies in Canadian childcare centres, while the provision of physical activity policies in other countries has shown some promise for improving the activity levels of young children. As such, the primary objective of the Childcare PhysicaL ActivitY (PLAY) Policy study is to examine the feasibility of an evidence-based, stakeholder-informed, written physical activity and sedentary time policy for centre-based childcare (i.e., at the institutional level). The secondary objectives are to examine the impact of policy implementation on the physical activity levels and sedentary time of young children, subsequent environmental changes in childcare centres, and childcare providers’ self-efficacy to implement a physical activity policy. This study will examine both policy implementation and individual (behavioural) outcomes. *Methods/Design*: The Childcare PLAY Policy study, a pilot, cluster-randomized controlled trial, involves the random allocation of childcare centres to either the experimental (*n* = 4) or control (*n* = 4) group. Childcare centres in the experimental group will adopt a written physical activity policy for eight weeks (at which time they will be asked to stop enforcing the policy). Physical activity levels and sedentary time in childcare will be assessed via ActiGraph^™^ accelerometers with measurements at baseline (i.e., week 0), mid-intervention (i.e., week 4), immediately post-intervention (i.e., week 9), and at six-month follow-up. Policy implementation and feasibility will be assessed using surveys and interviews with childcare staff. The Environment and Policy Assessment and Observation Self-Report tool will capture potential changes to the childcare setting. Finally, childcare providers’ self-efficacy will be captured via a study-specific questionnaire. A nested evaluation of the impact of policy implementation on young children’s physical activity levels will be completed. A linear mixed effects models will be used to assess intervention effects on the primary and secondary outcomes. Descriptive statistics and thematic analysis will be employed to assess the feasibility of policy implementation. *Discussion*: The Childcare PLAY Policy study aims to address the low levels of physical activity and high sedentary time observed in childcare centres by providing direction to childcare staff via a written set of evidence-informed standards to encourage young children’s activity and reduce sedentary time. The findings of this work will highlight specific aspects of the policy that worked and will inform modifications that may be needed to enhance scalability. Policy-based approaches to increasing physical activity affordances in childcare may inform future regulations and programming within this environment.

## 1. Background

Young children’s regular participation in physical activity positively impacts many aspects of health, including the maintenance of healthy body weight [1], cognitive development, and psychosocial and cardiometabolic health [2]. The recently released Canadian 24-Hour Movement Guidelines for the Early Years (0–4 years) provides recommendations regarding the appropriate amount and intensity of daily physical activity and sedentary time for young children [3]. Specifically, from age one, children are recommended to engage in 180 min/day of any intensity physical activity, including some moderate-to-vigorous physical activity (MVPA) [3]. Higher-intensity physical activity, in the form of energetic play, offers additional health benefits, and children three years and older should also engage in at least 60 min/day of MVPA [2,3]. Additionally, screen time should be restricted to no more than 60 min per day [3]. These new guidelines provide a target for parents and are especially important for childcare providers to consider, as more than half (60%) [4] of Canadian children under the age of six spend most of their waking hours enrolled in childcare services. Low levels of energetic physical activity have been reported in this setting (i.e., 1.5 min/h or approximately 12 min of MVPA during childcare hours) [5]; therefore, childcare-based physical activity interventions are necessary to ensure that children in this type of care are afforded sufficient opportunities to meet these guidelines.

Time spent in childcare is oftentimes sedentary [5,6,7,8,9]. Specifically, during childcare hours, Tucker and colleagues [9] noted that preschool-age children spend approximately 33 min/hour in sedentary pursuits, while Erinosho and colleagues reported 3.4 h/day sedentary among this population [7]. Additionally, childcare providers—the individuals responsible for the day-to-day care and programming within these centres—have noted low levels of self-efficacy for engaging children in physical activity [10], and the childcare environment, inclusive of portable play equipment and sedentary affordances, has been noted to influence physical activity opportunities and sedentary pursuits in these centres [5,9]. This is problematic as sedentary behaviours have been shown to negatively relate to motor/cognitive development, psychosocial health, and are linked to indicators of adiposity [11]. While some sedentary behaviours, such as reading, serve important developmental roles for young children, screen time has been noted as particularly detrimental among this cohort [11,12]. Many childcare centres have computers, tablets, and/or televisions available for children to use, and Vanderloo (2014) reported screen time use ranging from 0.1 to 1.3 hours per day in these facilities [12]. Replacing screen-based activities with physical activity affordances may support improved activity behaviours among young children in these settings.

A few studies, to date, have noted higher rates of physical activity participation among children in centres where an activity-specific or screen-viewing policy was in place [13,14,15,16,17]. However, as McWilliams and colleagues found in North Carolina [17], less than 60% of childcare centres had adopted a formal, written physical activity policy. Some of those policies that are in place included “vague statements such as ‘go outside daily, weather permitting’” [18]. Moreover, a number of researchers have noted the need for policies that offer clear guidance for childcare centres, along with specific methods for implementing the physical activity recommendations and tools for measuring the effectiveness of the strategy as well [6,19,20]. Tandon and colleagues further suggested that childcare physical activity policies should focus on outdoor play and adult-led physical activity, given their association with preschoolers’ activity levels [21], and Staiano et al. suggested limiting screen time and ensuring computer usage is for educational purposes only [16].

To gauge the current climate regarding childcare physical activity and screen time policies in Canada, our team recently examined the frequency of institutional-level policies and reviewed provincial/territorial regulations regarding physical activity and screen time [22,23]. At the individual childcare level, directors reported limited physical activity standards, and none explicitly defined a compulsory duration or intensity [22]. Similarly, of the 13 provinces and territories, only three referenced physical activity in their regulations, and of these, only two provide a time requirement for engaging children in physical activity [23]. Additionally, very few childcare centres noted having screen-based policies in place [22], and 92% of provincial/territorial regulations across the country have no stipulations specific to screen time in these settings [23]. Researchers have highlighted the need for more targeted research examining the impact of childcare policy changes on physical activity levels and sedentary time [24]. In Alberta and British Columbia, advances with respect to childcare accreditation standards have been made; specifically, in Alberta, accredited childcare centres are required to provide diverse daily offerings of physical activity, with childcare providers acting as role models for these activities [25]. A quasi-experimental evaluation of these accreditation standards supports the adoption of more specific guidelines to improve children’s physical activity levels and reduce sedentary time during care [26]. However, an examination of the effectiveness of an institutional childcare policy intervention outlining specific recommendations for physical activity among Canadian children has not yet been conducted.

The primary aim of the eight-week Childcare PLAY Policy study is to evaluate the feasibility of an evidence-based, stakeholder-informed, written physical activity and sedentary time policy in childcare centres. For the purpose of this study, feasible refers to the intervention being implemented as intended and well received by childcare providers. Specific objectives include determining whether the policy was effective at: (i) increasing young children’s (i.e., toddlers and preschoolers, age 18 months–4 years) physical activity (i.e., MVPA and total physical activity (TPA)); (ii) reducing young children’s sedentary time; (iii) producing a feasible and appropriate policy for use in the childcare setting; (iv) increasing childcare providers’ self-efficacy for facilitating young children’s physical activity during childcare; and (v) producing environmental changes in support of physical activity in childcare. Because the implementation of a policy may influence other aspects of the childcare, an environmental assessment will also be conducted. This pilot study will evaluate the feasibility and effectiveness of policy implementation within individual childcare centres, and it will afford the opportunity to modify the policy prior to examining its effectiveness in a larger, more diverse sample.

### Hypothesis

It is hypothesized that the physical activity and sedentary time policy will be viewed as feasible to implement. Additionally, it is anticipated that young children who attend a centre implementing the physical activity policy (i.e., experimental group) will display increased rates of TPA, MVPA, and reduced sedentary time from baseline to post-intervention, with no change observed for children at centres assigned to the control group. While activity levels are expected to decrease at follow-up, when providers are asked to stop enforcing the policy, it is believed that activity levels at this time point will still be higher than those recorded at baseline. For childcare providers from the experimental group, we expect an increase in self-efficacy implementing physical activity in childcare after adopting the policy and expect additional supportive changes in the childcare environment as a result of the policy implementation.

## 2. Methods

### 2.1. Study Design

In accordance with the Consolidated Standards of Reporting Trials statement (CONSORT) [27,28], a pilot, cluster-randomized controlled trial (RCT) will be conducted. Due to the nature of the intervention, a double-blind study design is not possible given that participants will be aware of their group designation, and as such, a single-blind design will be adopted (all assessments will be conducted by research staff who remain unaware of group assignment). This study received ethical approval from the University of Western Ontario’s Research Ethics Board (REB# 111890) and is registered with the Clinical Trials Registry provided by the US National Library of Medicine (NCT03695523; https://clinicaltrials.gov/).

### 2.2. Ethics Approval and Consent to Participate

Ethical approval was provided by the Health Sciences Research Ethics Board at The University of Western Ontario (REB# 111890). Parents/guardians of participating children provided written consent, and children offered assent when the accelerometer was placed on them. Childcare providers also provided written consent prior to participation.

### 2.3. Sample Size

To secure a representative sample of children in childcare, a random cluster sampling strategy will be used. In a meta-analysis exploring the effectiveness of 15 physical activity interventions targeting preschool-age children, Gordon and colleagues [29] reported that most studies had a small-to-moderate effect (Hedges *g* = 0.4) for change in young children’s total physical activity levels after participating in an intervention. If we convert this to f2, we can estimate this effect size to be approximately 0.12. Using the pwr package [30] within R version 3.6.0 [31], a minimum sample size of 99 children will be required (assuming 80% power and an experiment-wise alpha of 0.05). To account for a clustering effect, using an intra-cluster coefficient of 0.05, and an average cluster size of 16 children, the design effect is: 1 + 0.05 (16 − 1) = 1.75. Therefore, the sample size will be inflated to 175. Based on our recently conducted studies using accelerometry within the childcare environment [9], an anticipated loss to follow-up/accelerometer non-compliance rate of 20% is anticipated. To adjust for this, we aim to recruit 218 children.

### 2.4. Recruitment and Randomization

To secure an adequate sample size, this study will be conducted in eight randomly selected childcare centres (i.e., the clusters) chosen from an online listing of 55 eligible licensed childcare centres in London, Canada. Since it is necessary to implement the policy at the centre-level to groups of children, the childcare centres (i.e., the clusters) rather than the individual participants, will be randomly allocated by the project coordinator to either implement the physical activity policy (experimental; *n* = 4), or maintain their daily programming (control; *n* = 4) for the eight-week intervention period (at which time the policy will no longer be enforced).

### 2.5. Participants

The project coordinator will invite all toddlers (18 months–2.4 years) and preschoolers (2.5 years–4 years) to participate, and those whose parents/guardians provide written consent will be eligible to participate in the study. Childcare providers and directors from randomly selected childcare centres will also be invited to participate. Participants will receive a small token of appreciation as thanks for their time. See Table 1 for complete inclusion/exclusion criteria for the Childcare PLAY Policy study.

### 2.6. Development and Implementation of the Childcare PLAY Policy

To promote policy engagement, buy-in, and suitability, the policy was developed by our research team in collaboration with an advisory committee that included childcare organizations and centre administrators, childcare providers, municipal childcare stakeholders, and a policy expert. The policy was created following a review of relevant literature on the importance of outdoor play [32], unstructured activity [29,33], and informed by the Canadian 24-Hour Movement Guidelines for the Early Years (0–4 years; [3]). The current version of the policy includes eight statements that are designed to provide specific recommendations for time in energetic play (i.e., minimum of 40 min/day), outdoor time (i.e., 120 min/day), and suggests short (i.e., 15–30 min), frequent (i.e., 3–4 times/day) outdoor periods during childcare hours. It requires that children be exposed to a variety of indoor and outdoor physical activities to promote physical literacy and recommends appropriate role modelling of screen-based technology, with no screen time permitted for children during childcare hours. Sustained sedentary time is recommended to be disrupted and replaced with physical activity. See Appendix A for the Childcare PLAY policy.

Experimental Condition. For eight weeks, childcare providers in the experimental centres will be asked to implement the written policy that outlines optimal physical activity daily affordances and sedentary time recommendations during childcare. Prior to the eight-week intervention period, childcare providers will participate in a training session, led by the project coordinator. The session will last approximately an hour in length, which will detail the characteristics of the study design, review the policy, its implementation, and study tools (e.g., accelerometer logs).

Control Condition. Childcare centres randomly assigned to the control group will be asked to continue their typical daily curriculum for the duration of the eight-week intervention and follow-up period. Upon completion of the study, all centres allocated to this group may opt to receive a copy of the written physical activity policy and the training regarding its implementation.

### 2.7. Measures

A variety of tools will be used to assess the feasibility and effectiveness of the Childcare PLAY Policy (Table 2).

Demographic Information (Parents and Childcare Providers). Parents of participants will be asked to complete a demographic questionnaire at baseline to describe participants and their families (e.g., children’s age, sex, ethnicity, family income, parental education), as well as the children’s physical activity participation outside of childcare (e.g., organized sport or activities). Parental/guardian weekly engagement in MVPA and their view of themselves as a physical activity role model will be measured. Childcare providers and directors will complete a demographic questionnaire at baseline to capture information related to their age, sex, employment status, years of childcare experience, and level of education. Childcare providers will also report time in MVPA and their view of themselves as physical activity role models. No personal identifiers will be stored with participant data.

Physical Activity (Children). Toddlers’ and preschoolers’ physical activity will be measured using ActiGraph™ (ActiGraph, LLC, Pensacola, Florida) accelerometers—the gold standard for measuring young children’s sporadic physical activity patterns in field settings [34,35,36]—for five consecutive days, during childcare hours only. This process will be undertaken pre-intervention (week 0), mid-intervention (week 4), post-intervention (week 9), and at six-month follow-up. Raw data will be collected to capture the sporadic activity and intermittent periods of rest of the young participants. The accelerometers will be worn on the right hip (above the iliac crest) and will begin collecting activity data on the morning of the first day of data collection. Wear-time of the accelerometers (i.e., the time when it is fitted and removed from each child) will be recorded for each participating child by childcare providers using a daily log. Validated and age appropriate cut-points will be applied to the data to delineate the different activity intensities [37].

Anthropometric Measures (Children). Height (using a Seca 214 “Road Rod” Portable Stadiometer; nearest 0.1 cm), weight (using a Tanita 700-TBF300GS Body Fat Analyzer w/Goal Setter scale; nearest 0.1 kg), and waist circumference (using a measuring tape; nearest 0.1 cm) will be assessed at baseline. The data collected will be used to calculate the child’s standardized body mass index score (BMI-z).

Policy Adherence Log (Childcare Providers). A log will be completed by childcare providers three times per week (i.e., Monday, Wednesday, and Friday) to monitor adherence to each statement of the policy during the eight-week intervention period. Childcare providers will check “yes” to indicate that they adhered to the policy statement, “part” to indicate that they only partially followed the policy item, and “no” to indicate that the policy statement was not followed on that particular day. Reasons the policy was not followed will also be indicated from a list of options (i.e., weather, ratios, no space, behaviour, other).

Self-Efficacy (Childcare Providers). To gauge potential changes in childcare providers’ self-efficacy to provide physical activity opportunities and overcome related barriers, a 40-item instrument was developed for the purpose of the study, which will be administered twice at baseline (to assess test, re-test reliability) and at all subsequent time points. Childcare providers will rate their confidence to each item on a scale from 0 (I am not at all confident) to 10 (I am highly confident) in line with the construction of self-efficacy scales (e.g., task and barrier) [38].

Program Evaluation Survey (Childcare Providers). Childcare providers from the experimental condition will complete a survey (Appendix C) at post-intervention to gather information to evaluate implementation of the policy. Specifically, their initial willingness to participate (one item), preparedness (one item), and the feasibility or ease of policy implementation (18 items), will be rated on a scale from 1 (strongly disagree) to 5 (strongly agree). Future implementation will be assessed via 17 items rated from 1 (not at all likely) to 5 (extremely likely), and communication (four items) will be rated from 1 (not at all effective) to 5 (extremely effective). Nine open-ended response questions will capture aspects of the policy that the childcare providers liked the most, those that they found to be most/least important, challenges encountered, solutions used, any modifications made, the noted success of modifications, and their overall experience with policy implementation. The tool was created for the purposes of this study.

Interviews (Childcare Providers). As this is a pilot study, interviews will be completed with interested childcare providers (*n* ≈ 8–10) from the experimental condition. Using a semi-structured interview guide (Appendix B), these in-depth discussions will provide a greater understanding of the appropriateness of the policy, the feasibility of implementation, and suggestions for improvements that add to what is captured in the program evaluation survey. Following implementation of the eight-week policy intervention, childcare providers will be invited to participate in an interview (in person or via telephone), which will be audio recorded and transcribed verbatim.

Childcare Environment (Childcare Providers and Directors). The validated Environment and Policy Assessment and Observation—Self-Report (EPAO-SR; [39]) instrument examines the classroom physical environment and physical activity practices in childcare centres; this tool consists of two subscales, Space, Equipment, and Environment (eight items) and Practices around Physical Activity (three items). We will adapt the tool to conform with the Canadian context and remove the nutrition content (as seen in Ott et al.’s paper) [22]. This tool will be administered to childcare providers once during each week of data collection. The information solicited from this questionnaire will provide useful context to help better understand each classroom’s physical activity environment and practices and whether these change after the policy is implemented. The EPAO-SR Today [39], which entails six subscales (Morning Outdoor Activities (seven items); Morning Indoor Activities (eight items); Nap/Rest Time (three items); Afternoon Outdoor Activities (seven items); Afternoon Indoor Activities (three items); and Other Activities (three items)) will also be completed by one childcare provider per classroom on one chosen day at each data collection time point. This validated tool provides a snapshot of the specific activities in which the children engaged during morning and afternoon indoor and outdoor periods. Finally, the validated Director General EPAO-SR [39], which consists of two subscales (Childcare Environment (six items) and Physical Activity Policies (11 items)) also adapted for use in Canada without the nutrition subscale, will be administered to all centre directors at baseline. This will assess the presence of any existing physical environment characteristics that may inhibit or promote physical activity, and existing policies related to physical activity and sedentary time that may exclude a centre from participating in the study.

### 2.8. Analysis

All data will be entered, verified, and stored in our lab. Baseline characteristics of the children and the childcare centres will be summarized descriptively; participant characteristics for those who complete the study and those who drop out will be explored. All statistical analyses will be performed in R [31], using the lme4 [40] and lmerTest packages [41].

Implementation Log. Frequencies and percentage scores will be calculated to reflect adherence to policy statements and reasons that compliance was not attained will be reported descriptively.

Program Evaluation Survey and Interviews. Means and standard deviations of survey item ratings will be provided. Responses to open-ended program evaluation survey and interview questions will be analyzed using thematic content analysis [42] using QSR NVivo.

Physical Activity. Children’s physical activity data will be analyzed using a series of linear mixed effects models for each of the three outcome variables (i.e., TPA, MVPA, and sedentary time). Group (experimental versus control) and time (baseline, mid-intervention, post-intervention, and six-months post-intervention) will be modelled as a fixed effect, and will be evaluated using *t*-tests, with a Satterthwaite approximation determining degrees of freedom. Descriptive survey data will be analyzed using frequencies, *t*-tests, and chi-square tests.

Childcare Providers’ Self-Efficacy. Descriptive statistics will be reported to indicate changes in childcare providers’ self-efficacy specific to implementing physical activity in childcare. Classical test theory will be used to assess internal consistency of the scales, exploratory factor analysis will be used to assess the factorial validity of the measured constructs, and item response theory will be used to explore the item and scale characteristics of the measurements used to assess the two polytomous constructs in this study (i.e., task and barrier self-efficacy). Factor analysis and classical test theory analyses will be undertaken within the psych package [43] and the ltm package [44] will be used for the item response theory analyses.

Childcare Environment. The EPAO-SR is structured around 13 best-practices scores which are obtained from the three surveys [39]. The revised EPAO-SR tools will be scored, in line with the scoring protocols as described by the tool creators [39], to generate a score for each of the 13 best practices. Descriptive statistics will be used to describe classroom practices and changes in classroom practices and environment will be explored using 2 × 5 logistic regression models (two groups, five time points).

## 3. Discussion

Evidence-informed approaches to supporting and encouraging appropriate physical activity participation (and limiting periods of sedentary time) during childcare hours are warranted. A substantial body of evidence suggests that childcare programming and the environment strongly influence young children’s physical activity levels [45,46]. In fact, childcare centres influence preschoolers’ activity levels more than individual-level factors, such as age, sex, and ethnicity [47]. Despite recognition that childcare venues are an ideal setting to intervene and the call for institutional-level physical activity policies in childcare centres [23], the feasibility and effectiveness of a physical activity and sedentary time policy, specifically within the Canadian context, is lacking. A written physical activity and sedentary time policy may help to promote providers’ self-efficacy by providing specific direction in terms of how to best engage children in daily activity. Moreover, creating and implementing such policies would ensure that all children attending these facilities are afforded the opportunity to be physically active, and offer a greater chance of meeting the daily recommendation. Young children spend two-thirds of their day in childcare, suggesting that two-thirds of the daily movement requirement (180 mins of TPA; 60 mins of MVPA), may be achieved during childcare hours (120 mins of TPA; 40 mins of MVPA). Implementation of an activity-focused childcare policy provides an opportunity to “level the playing field” in terms of ensuring consistent affordances within this setting and represents a promising population-based approach (with access to over 50% of this Canadian cohort) for supporting active behaviours.

While Alberta has demonstrated that new accreditation standards show promise in improving activity levels among young children [26], this research will be the first to explore the feasibility and effectiveness of an institutional-level written childcare policy intervention as a mechanism to support improved physical activity levels (and minimize sedentary time) among young children in Ontario. By providing specific, evidence-informed guidance that offers strategies for achieving success at increasing physical activity and reducing sedentary time, childcare providers will be in a better position to ensure their programming is in line with Canadian recommendations [48]. Moreover, this study will offer an understanding of the uptake and receptivity of childcare centres to implementing such a policy, and feasibility and the burden of doing so. If effective, and appropriate to implement, the findings from the Childcare PLAY Policy study have the potential to guide institutional-level policies (short-term) and higher-level regulatory amendments to provincial standards (long-term). The effectiveness of the intervention rests in the creation of a physical activity policy that childcare providers believe is important and feasible to implement and aligns with their current curriculum and pedagogy. The results of the Childcare PLAY Policy will be shared with participants and childcare professionals and stakeholders via publications, infographics, and presentations.

## Figures and Tables

**Table 1:** Inclusion and exclusion criteria for the Childcare PLAY Policy study.

Participant	Inclusion Criteria	Exclusion Criteria
Childcare Centres	Centre-based facility in London, Canada At least one toddler or preschool classroom Childcare providers willing to participate ≥8 children who received parental consent English-speaking centre Does not currently have an institutional-level PA policy	Home-based childcare or after-school care only Not located in London, Canada or surrounding area Do not have a toddler or preschool-age classroom Childcare providers not willing to participate <8 children with parental consent Not an English-speaking facility Already has an established institutional-level PA policy
Childcare Providers	Full-time childcare provider in a toddler/preschool classroom Speaks and writes English	Not full-time Not a childcare provider in a toddler/preschool classroom Does not speak/write in English
Toddler/ Preschool Participants	Enrolled at a participating childcare centre 18 months–4 years Expected to remain at centre for next eight months Enrolled in a toddler/preschool classroom Parent/guardian speaks and understands English Has parental/guardian consent	Not enrolled at a participating childcare centre Not 18 months–4 years Not expected to remain in care for next eight months Not enrolled in a toddler/preschool classroom Parent/guardian does not read/write in English No parental/guardian consent

*Note.* PA = physical activity.

**Table 2:** Outcome assessments for the Childcare PLAY Policy study.

Participant/Tool		Control Condition				Experimental Condition			
		Baseline (week 0)	Mid-Int (week 4)	Post-Int (week 9)	6M	Baseline (week 0)	Mid-Int (week 4)	Post-Int (week 9)	6M
Toddlers/ Preschoolers	Parent/Guardian Demographic Q	x				x			
	Physical Activity/ Sedentary Time (ActiGraph™ data)	x	x	x	x	x	x	x	x
	Anthropometrics	x				x			
Childcare Providers	Demographic Q	x				x			
	Policy Adherence Log (week 1–8) *		x				x		
	Program Evaluation Survey							x	
	Interview							x	
	EPAO-SR General	x	x	x	x	x	x	x	x
	EPAO-SR Today	x	x	x	x	x	x	x	x
	Self-Efficacy Q	x	x	x	x	x	x	x	x
Directors	Director General EPAO-SR	x				x			

*Note*. Mid-Int = mid-intervention, Post-Int = post-intervention, M = month, Q = questionnaire, EPAO-SR = Environment and Policy Assessment and Observation Tool—Self-Report Tool. * Policy Adherence Log was maintained for the duration of the eight-week intervention, but not at baseline or post-intervention/follow-up measurements.

## Abbreviations

CONSORT: Consolidated Standards of Reporting Trials; RCT: randomized controlled trial; TPA: total physical activity; MVPA: moderate-to-vigorous physical activity; REB: research ethics board; BMI: body mass index; EPAO-SR: Environment and Policy Assessment and Observation—Self-Report.

## Appendix A. Childcare PLAY Policy

Childcare programs encourage all children to engage in physical activity frequently throughout the day, with a focus on outdoor energetic free play and deliberate interruption of sustained periods of sedentary behaviour.

Directed by the Canadian 24-Hour Movement Guidelines for the Early Years (0–4 years)*, childcare programs are expected to:

1. Encourage children to engage in higher intensity energetic play (i.e., activities that induce sweating and heavy breathing) often throughout the day with a goal of accumulating a minimum of 40 min each day. More is better.
2. Expose children to a variety of indoor and outdoor physical activities, including both child-directed and teacher-facilitated active play daily.
3. Outdoor time is offered for a minimum of 120 min each day, unless extreme weather (i.e., heat or cold alert) prevents it. When extreme weather occurs, the opportunity exists for children to engage in active play indoors.
4. Short, frequent outdoor sessions are most conducive to higher intensity physical activity among children; therefore, short bouts (e.g., 15–30 min) of outdoor time are recommended often (e.g., 3–4 times a day).
5. Unstructured (i.e., child-directed) free play is predominant during outdoor time. When activity levels decline, childcare practitioners encourage continued energetic play through structured activity, participation alongside children, and use of verbal prompts.
6. Encourage children to develop physical literacy by practicing fundamental movement skills often throughout the day (e.g., running, skipping, hopping, or jumping).
7. The appropriate use of screen-based technology is role modelled by childcare practitioners by avoiding it when children are present. Screen-based technology is not offered to children under 2 and is not recommended during childcare hours.
8. Programming is designed to break up sustained sedentary time using indoor movement-based activities.

* These guidelines recommend that children over 1 year of age engage in 180 min of physical activity at any intensity each day, and by age 3, at least 60 min of this time is spent in higher intensity physical activity, known as energetic or active play.

## Appendix B. Semi-Structured Interview Guide

Thank you for volunteering to participate in this interview. We are here today to discuss your thoughts on the recently implemented Childcare PLAY policy intervention; a physical activity policy targeting toddlers and preschoolers in centre-based childcare. Specifically, we are looking to gather your feedback on the feasibility of introducing this policy into childcare facilities. Your feedback on this topic is important. There are no right or wrong answers.

1. Overall, what has been your **overall experience** with implementing the physical activity policy?
  - How ‘feasible’ (i.e., convenient and easy) was this policy to implement?
  - How receptive were staff to implementing this policy?
  - Does anyone have anything else to add?
2. What were the **best** parts of the policy?
  - What made those parts/characteristics so beneficial?
  - What are some examples of these?
  - Tell me more about that.
3. What characteristic(s) of the policy do you feel was/were **most** appropriate for increasing physical activity participation among the children in your care?
  - What made it/them so appropriate?
  - What are some examples?
  - Who else experienced something similar? Who experienced something different/in contrast?
  - How ‘effective’ would you consider this policy in increasing children’s physical activity levels during childcare hours?
4. What characteristic(s) of the policy do you feel was/were **least** appropriate for increasing physical activity participation among the children in your care?
  - What made it/them so inappropriate?
  - What are some examples?
  - Who else experienced something similar? Who experienced something different/in contrast?
  - How do you think this aspect of the policy could be tweaked so that it is more appropriate for the childcare environment?
5. What **challenges** did you experience when implementing the policy?
  - Please expand.
  - In what ways did this impact the implementation of the policy?
  - How well did you implement the policy?
6. What **solutions** did you undertake to deal with these challenges?
  - Please expand.
  - Tell me more about that.
  - How much time and effort did these solutions require?

## Appendix C. Program Evaluation Survey

We appreciate the time and effort you have put into implementing the childcare physical activity policy. To gain a better understanding of the feasibility of policy implementation, as well as the appropriateness of the policy components, please respond to the following questions. It should take approximately 10 minutes to complete this survey. Your feedback will serve as an important first step in the evaluation of the PLAY childcare physical activity policy. More specifically, your comments will inform potential modifications to the PLAY policy for use in the future. All results collected from this survey will remain confidential and anonymous.

Instructions: Please *circle* the number that best corresponds with your response to the following questions.

### SECTION 1: FEASIBILITY (i.e., ease of implementation) OF POLICY IMPLEMENTATION

1. Please, rate the degree to which you agree or disagree with the following statements about the PLAY policy.

	**Strongly Disagree**		**Neither Agree or Disagree**		**Strongly Agree**
a. When first approached to participate, I was very receptive to implementing the PLAY policy.	1	2	3	4	5
b. I felt adequately prepared to implement the PLAY policy.	1	2	3	4	5
c. The PLAY policy was easy to implement.	1	2	3	4	5
d. It was not easy to encourage children to engage in physical activity frequently throughout the day.	1	2	3	4	5
e. It was easy to encourage children to engage in higher intensity energetic play frequently throughout the day.	1	2	3	4	5
f. It was easy to provide children with the opportunity to achieve a minimum of 40 minutes of higher intensity energetic play each day.	1	2	3	4	5
g. It was not easy to expose children to a variety of indoor physical activities each day.	1	2	3	4	5
h. It was easy to expose children to a variety of outdoor physical activities each day.	1	2	3	4	5
i. It was easy to provide unstructured or child-directed free play each day.	1	2	3	4	5
j. It was not easy to provide structured or teacher-facilitated active play each day.	1	2	3	4	5
k. It was easy to offer a minimum of 120 minutes of outdoor time each day.	1	2	3	4	5
l. It was easy to provide the opportunity for children to engage in active play indoors when outdoor play was not possible.	1	2	3	4	5
m. It was not easy to provide shorter, more frequent outdoor play sessions.	1	2	3	4	5
n. It was easy to encourage continued energetic play through structured or teacher-led activities.	1	2	3	4	5
o. It was easy to encourage continued energetic play through teacher participation in physical activity.	1	2	3	4	5
p. It was not easy to encourage continued energetic play using verbal prompts.	1	2	3	4	5
q. It was easy to support children’s development of physical literacy through encouragement of fundamental movement skills (e.g., running, skipping, hopping, or jumping).	1	2	3	4	5
r. It was easy to avoid using my own screen-based technology when the children were present.	1	2	3	4	5
s. It was easy to avoid children’s exposure to screen-based technology during childcare hours.	1	2	3	4	5
t. It was not easy to break up children’s sedentary time by providing indoor active play opportunities.	1	2	3	4	5

### SECTION 2: FUTURE IMPLEMENTATION

1. Although the formal implementation of the PLAY policy has come to an end, how likely are you to continue to adopt the following aspects of the policy within your classroom?

**I Plan to Continue…**	**Not at All Likely**		**Somewhat Likely**		**Extremely Likely**
a. to encourage children to engage in physical activity frequently throughout the day.	1	2	3	4	5
b. to encourage children to engage in higher intensity energetic play often throughout the day.	1	2	3	4	5
c. to provide children with the opportunity to achieve a minimum of 40 minutes of higher intensity energetic play each day.	1	2	3	4	5
d. to expose children to a variety of indoor physical activities each day.	1	2	3	4	5
e. to expose children to a variety of outdoor physical activities each day.	1	2	3	4	5
f. to provide unstructured or child-directed free play each day.	1	2	3	4	5
g. to provide structured or teacher-facilitated active play each day.	1	2	3	4	5
h. to offer a minimum of 120 minutes of outdoor time each day.	1	2	3	4	5
i. to provide the opportunity for children to engage in active play indoors when outdoor play is not possible.	1	2	3	4	5
j. to provide shorter, more frequent outdoor sessions.	1	2	3	4	5
k. to encourage continued energetic play through structured or teacher-led activities.	1	2	3	4	5
l. to encourage continued energetic play through teacher participation in physical activity.	1	2	3	4	5
m. to encourage continued energetic play through verbal prompts.	1	2	3	4	5
n. to support children’s development of physical literacy through the encouragement of fundamental movement skills (e.g., running, skipping, hopping, or jumping).	1	2	3	4	5
o. to avoid my own use of screen-based technology when children are present.	1	2	3	4	5
p. to avoid children’s exposure to screen-based technology during childcare hours.	1	2	3	4	5
q. to break up children’s sedentary time by providing indoor active play opportunities.	1	2	3	4	5

### SECTION 3: COMMUNICATION

1. With regard to the planning and implementation of the PLAY policy, how effective did you feel the communication was between the following?

**How Effective Was the Communication…**	**Not at all Effective**		**Somewhat Effective**		**Extremely Effective**
a. between the research team and your centre?	1	2	3	4	5
b. between your director and the staff?	1	2	3	4	5
c. between and among staff members?	1	2	3	4	5
d. between staff and/or the director and parents?	1	2	3	4	5

### SECTION 4: GENERAL THOUGHTS ABOUT THE PLAY POLICY

1. What did you like most about the PLAY policy?
2. What part of the PLAY policy did you feel was most important?
3. What challenges did you experience when implementing the PLAY policy?
4. What solutions helped you to resolve these challenges?
5. During the intervention period, were there any aspects of the policy that you modified? Please describe
6. If you made modifications, were they successful?
7. If you could modify the PLAY policy in any way, what would you change? Why?
8. Did you observe any changes in the children’s moods, or behaviour when implementing the PLAY policy?
9. What else do you want us to know about your experience with the PLAY policy?
